# Efficacy and Safety of Postbiotic Contained Inactivated *Lactobacillus reuteri* (*Limosilactobacillus reuteri*) *DSM*17648 as Adjuvant Therapy in the Eradication of *Helicobacter pylori* in Adults With Functional Dyspepsia: A Randomized Double-Blind Placebo-Controlled Trial

**DOI:** 10.14309/ctg.0000000000000750

**Published:** 2024-07-18

**Authors:** Vladimir Ivashkin, Igor Maev, Elena Poluektova, Alexander Sinitsa, Elena Avalueva, Marina Mnatsakanyan, Vladimir Simanenkov, Julia Karpeeva, Daria Kopylova, Irina Kuprina, Yury Kucheryavyy, Tatiana Lapina, Olga Solovyeva, Malle Soom, Natalia Cheremushkina, Evgeniya Maevskaya, Roman Maslennikov

**Affiliations:** 1 Institute of Clinical Medicine, Sechenov First Moscow State Medical University (Sechenov University), Moscow, Russia;; 2 Department of Propedeutics of Internal Diseases and Gastroenterology, A.I. Yevdokimov Moscow State University of Medicine and Dentistry, Moscow, Russia;; 3 Scientific Department, The Interregional Public Organization “Scientific Community for the Promotion of the Clinical Study of the Human Microbiome”, Moscow, Russia;; 4 Scientific Department, «Kraft Group», Saint Petersburg, Russia;; 5 Gastroenterology Department, Federal State Establishment Clinical Diagnostic Medical Centre, Saint Petersburg, Russia;; 6 Department of Internal Medicine, Clinical Pharmacology and Nephrology, North-Western State Medical University named after I.I. Mechnikov, Saint Petersburg, Russia;; 7 Gastroenterology Department, Voronezh Regional Clinical Hospital No. 1, Voronezh, Russia;; 8 Gastroenterology Department, Ilyinskaya Hospital, Glukhovo, Russia;; 9 Scientific Department, Parusin GmbH, Hamburg, Germany.

**Keywords:** postbiotics, metabiotics, functional digestive disease, probiotics, microbiota

## Abstract

**INTRODUCTION:**

Increasing the effectiveness of eradication therapy is an important task in gastroenterology. The aim of this study was to evaluate the efficacy and safety of postbiotic containing inactivated (nonviable) *Limosilactobacillus (Lactobacillus) reuteri DSM*17648 (Pylopass) as adjuvant treatment of *Helicobacter pylori* eradication in patients with functional dyspepsia (FD).

**METHODS:**

This randomized, double-blind, placebo-controlled, multicenter, parallel study included *H. pylori*-positive patients with FD. The postbiotic group received Pylopass 200 mg bid for 14 days in combination with eradication therapy (esomeprazole 20 mg bid + amoxicillin 1,000 mg bid + clarithromycin 500 mg bid for 14 days) and another 14 days after the completion of eradication therapy. The study was registered in the ISRCTN registry (ISRCTN20716052).

**RESULTS:**

Eradication efficiency was 96.7% for the postbiotic group vs 86.0% for the placebo group (*P* = 0.039). Both groups showed significant improvements in quality of life and reduction of most gastrointestinal symptoms with no significant differences between groups. The overall number of digestive adverse effects in the postbiotic group was lower than in the placebo group. Serious adverse effects were not registered.

**DISCUSSION:**

The postbiotic containing inactivated *L. reuteri DSM*17648 significantly improves the effectiveness of *H. pylori* eradication therapy in FD and decreases overall number of digestive adverse effects of this therapy.

## INTRODUCTION

According to the Rome IV criteria, functional dyspepsia (FD) is a complex of symptoms (postprandial fullness, early satiation, epigastric pain, and epigastric burning) for the last 3 months with symptom onset at least 6 months before diagnosis with no evidence of organic, systemic, or metabolic disease that would explain the symptoms on routine investigations (1,2). According to the results of large-scale studies, the prevalence of FD worldwide ranges from 10 to 30%. Patients with FD have a reduced quality of life, which is associated with the severity of symptoms and concomitant depression, the need for high medical costs, and reduced ability to work (1,3). The pathogenesis of FD development is not fully understood (1–4).

The prevalence of *Helicobacter pylori* among patients with FD ranges from 20% to 60% (5). Eradication therapy in these patients led to a decrease in the symptom severity in some patients, but it is not always effective (1,6,7). To increase the efficacy of eradication therapy, several methods have been proposed, including addition of probiotics and mucosal protective agents such as rebamipide to standard eradication therapy. This complementation showed a positive effect (6–9).

The *Lactobacillus reuteri* (*Limosilactobacillus reuteri* according to the most current classification) strain *DSM*17648 (DSM - Deutsche Sammlung von Mikroorganismen [German Collection of Microorganisms]) was selected among 700 strains of *Lactobacillus* because it demonstrated highly specific antagonistic binding to *H. pylori* cells in artificial gastric juice, reducing their mobility and adhesion ability. Only 7 other strains of *Lactobacillus* had this effect, but all of them had autoaggregation properties, which significantly reduced their anti-Helicobacter activity. *L. reuteri* strain *DSM*17648 coaggregates with different types and species of *Helicobacter* (*H. pylori* type I and II, *H. heilmannii* type I and II, *H. canis*) but not with bacterial representatives of normal oral or intestinal microbiota (10).

Recent studies have shown that not only living, but also inanimate microorganisms, and/or their components (postbiotics, also called metabiotics, ghostbiotics, or paraprobiotics) can confer health benefits (11,12). Nonviable microorganisms exert their positive effect due to metabolites, signaling and structural molecules, which preserve their biological activity after inactivation of the cells. For example, surface proteins of both living and inanimate bacteria can bind target bacteria (e.g., *H. pylori*). In addition, intracellular metabolites that exit damaged microbial cells can have a stimulating, inhibitory, and modulating effect on other microorganisms and tissues of the macroorganism. The exact mechanisms of action of postbiotics remain to be clarified (11,12). Postbiotics are safer for consumers, faster in action, and less demanding to storage conditions than probiotics because they do not contain living bacteria. Postbiotic based on *L. reuteri DSM*17648 with a trade name Pylopass reduced the activity of *H. pylori*, and prevented secondary diseases and symptoms related to *H. pylori* infection. Moreover, the preventive effect was carried over well after the supplementation itself, at least for the 6-month period (10,13,14). However, there has been no multicentered placebo-controlled randomized study so far directly showing the ability of Pylopass to increase the effectiveness of eradication therapy in adults. Evaluation of the efficacy and safety of postbiotic based on *L. reuteri DSM*17648 as a supplementation for the *H. pylori* eradication therapy in patients with FD was the aim of this study.

## METHODS

This randomized, double-blinded, placebo-controlled, multicenter, parallel study was approved by the Local ethics committee (protocol № 10–19 dated July 17, 2019) in accordance with the Helsinki declaration and registered in the International Standard Randomised Controlled Trial Number (ISRCTN) registry (ISRCTN20716052).

Randomization lists were created using the blockrand package (version 1.3) of the Microsoft R Open statistical software with the following parameters: 2 blinded groups with equal distribution into groups; 6 uniformly created randomization lists—1 for each center; randomization sequences (seed) were 4,037 (Center 1), 3,031 (Center 2), 3,739 (Center 3), 3,034 (Center 4), 3,259 (Center 5), and 3,125 (Center 6); variable block length (2, 4, 6, 8, 10, 12, 14, 16, 18, or 20), total number of blocks were 18 for Center 1, 17 for Center 2, 16 for Center 3, 16 for Center 4, 20 for Center 5, and 18 for Center 6.

### Patients

All patients who visited the clinics of the centers with characteristic complaints of FD were screened for participation in the study. The inclusion criteria were male or female aged 18–65 years, mandatory use of contraceptive methods for woman of childbearing age, confirmed diagnosis of FD according to the Rome IV criteria (1), presence of *H. pylori* infection according to 13C urease breath test (UBT) (6,7), no history of previous eradication therapy at least a year before the screening, ability to understand and willingness to follow all protocol details, and signed informed consent.

The exclusion criteria were erosive, ulcerative, or cicatricial changes in the stomach and/or duodenum; history of eradication therapy <1 year before screening; use of antibiotics and/or bismuth trication dicitrate and/or H2 secretion blockers and/or proton-pump inhibitors 30 days before and during the study; use of macrolide antibiotics <1 year before screening; any severe, decompensated, or unstable medical condition that could affect the clinical evaluation of the investigational product or put the patient at risk; pregnancy, lactation; known sensitivity to any components of the study product, and any of the drugs prescribed in this study; history of surgical treatment of the stomach, resection of the small intestine or operations on the pancreas; a positive blood test result for HIV and/or syphilis and/or hepatitis B surface antigen (HBsAg) and/or anti-hepatitis C virus antibody (HCV-Ab); chronic diarrhea of various etiologies, except for functional diarrhea or irritable bowel syndrome with diarrhea; participation in another clinical study 30 days before and during the study; use of probiotics, symbiotics, and prebiotics for the treatment of *H. pylori* infections and for other reasons within 30 days before study entry; refusal to continue the study, including the refusal of visits 3 and/or 4 and the investigations on these visits (other than taking blood for analyses); development of a clinical condition that is associated with safety and, in the opinion of the investigator, requires termination of participation in the clinical study; failure to comply with the minimum duration of eradication therapy (10 days, compliance <75%); and use during the treatment and follow-up period of antibiotics, probiotics, prebiotics, postbiotics, antisecretory or bismuth drugs, sucralfate, drugs with a pronounced hepatotoxic and nephrotoxic effect, with the exception of drugs that were used in the tested eradication therapy regimens.

Based on the study by Zagari et al (15), for sample size estimation, we initially assumed that the eradication rate would be 75% in the control group and 90% in the postbiotic group, which at significance level of 95%, power of 80%, and randomization rate of 1:1 gave the required number of included patients of 172 persons. However, the development of the coronavirus disease 2019 (COVID-19) pandemic has slowed down the patient enrollment. Therefore, we revised our calculations according to a more recent work by De Francesco et al (16), estimating the eradication rate of 79% in the control group and 94% in the postbiotic group. Based on these data, we calculated the required number of patients with STATISTICA 10 software (StatSoft Inc., Tulsa, OK). It was estimated as 64 patients for each group which corresponds to 128 included patients.

### Intervention

Patients were randomized 1:1 to the postbiotic and placebo groups. Out-patients in the postbiotic group received 1 capsule with 324 mg Helinorm containing 200 mg Pylopass (2 × 10^10^ spray-dried *L. reuteri DSM*17648 cells) bid with meals for 14 days together with standard eradication therapy (esomeprazole 20 mg bid 15–30 minutes before meals + amoxicillin 1,000 mg bid after meal + clarithromycin 500 mg bid after meal for 14 days) and another 14 days after completion of eradication therapy.

Pylopass is produced by fermentation of *L. reuteri* strain *DSM*17648. These bacteria are grown in a specific medium, inactivated, and dried using a patented technology. Pylopass specifically coaggregates *H. pylori*. It is proposed that this interferes with mobility and adherence of *H. pylori* to gastric mucosa. Aggregated *H. pylori* no longer adhere to the epithelium and are flushed from the stomach by natural bowel movement (13). Postbiotic dosage was chosen based on the previous experimental data (17).

Helinorm is a registered trade name of a dietary supplement in the country where this study was conducted, which contains Pylopass and neutral substances used to create the dosage form. Helinorm and placebo had an identical composition of neutral formative substances (117.5 mg of lactose and 6.5 mg of E551 per capsule). Instead of Pylopass, the placebo contained 200.0 mg of corn dextrin Nutriose FM06. Placebo and Helinorm capsules were indistinguishable from each other in appearance, smell, and taste.

### Controls

Out-patients in the placebo (control) group received placebo at a dose of 324 mg bid with meals for 14 days simultaneously with standard eradication therapy (esomeprazole 20 mg bid 15–30 minutes before meal + amoxicillin 1,000 mg bid after meal + clarithromycin 500 mg bid after meal for 14 days) and another 14 days after completion of eradication therapy. Visually, the placebo capsules and their jars did not differ from the postbiotic capsules and jars. Neither the patients nor the medical staff working with them knew whether it was the postbiotic or the placebo.

### Outcomes

The primary outcome was successful eradication of *H. pylori*, defined as negative 13С-UBT 28 days after the end of the postbiotic/placebo administration (42 days after the end of eradication therapy). Urease breath tests are currently considered to be the golden standard for diagnosis of *H. pylori* infection (6). The period of eradication therapy (14 ± 2 days) was defined as the treatment period, and the period after the end of eradication therapy until the repeated breath test for *H. pylori* (42 ± 3 days) was defined as the follow-up period.

13C-UBT was carried out according to the same methodology at the inclusion of the study and at its end. It was performed in the morning on an empty stomach using the Helicarb kit (Isocarb Limited, Moscow, Russia). The patient drank 200 mL of orange juice included in this kit, and 5 minutes after it, he/she exhaled air into the urease test bag 1. Then, he/she was given a 50 mL aqueous solution containing 50 mg of 13C-urea. During the next 30 minutes, the patient did not eat, rested, and did not smoke, and subsequently, a new sample of exhaled air was taken from him/her in the urease test bag 2. Both bags were delivered to the laboratory on the same day or in the morning of the next day, where their contents were analyzed by infrared isotope analyzer IRIS-Doc (Kibion AB, Sweden) in accordance with the manufacturer's instructions. The presence of *H. pylori* infection was assessed by an increase in 13C-carbon dioxide between the samples UBT 2 and UBT 1, which these bacteria produce from the 13C-urea with the urease enzyme in the stomach.

Secondary outcomes included (i) changes in the severity of digestive symptoms, defined as the changes in the 7 × 7 (18) and Gastrointestinal Symptom Rating Scale (19) questionnaire scores; (ii) changes in the quality of life, defined as the changes in the Short Form 36 (SF-36) (20) questionnaire scores; (iii) changes in the value of the main parameters of a complete blood count (hemoglobin, white blood cell and platelet count, erythrocyte sedimentation rate) and a biochemical blood test (the serum levels of total protein, total bilirubin, creatinine, amylase, alkaline phosphatase, alanine transaminase, and aspartate aminotransferase); (iv) frequency and severity of adverse effects of the therapy; and (v) therapy compliance, defined as the ratio of the number of used drugs to those that should be used. The number of used drugs was estimated by the number of empty places in the drug blisters, which patients returned by the end of eradication therapy. The study scheme is presented in Table 1.

**Table 1.** Study scheme

	Screening	Eradication treatment		Posteradication treatment	Follow-up
		14 d		14 d	28 d
	Visit 1 (day −8 –[-1])	Visit 2 (day 0)	Visit 3 (day 14)		Visit 4 (day 56 ± 3)
Postbiotic/Placebo taking					
Eradication treatment					
Demographics	+				
Medical history	+				
Complete blood count	+		+		
Serum chemistry	+		+		
Anti-HIV Ab, RPR, HBsAg, HCV-Ab	+				
Gastroscopy	+				
С-13 urease breath test for *Helicobacter pylori*	+				+
Pregnancy test	+				
Signing informed consent	+				
Checking inclusion and exclusion criteria		+	+		+
Randomization		+			
Initiation of eradication therapy for 14 d + postbiotic/placebo for 28 d		+			
7 × 7 Questionnaire		+	+		+
GSRS questionnaire		+	+		+
SF-36 questionnaire		+	+		+
Assessment of adverse effects			+		+
Compliance assessment			+		

GSRS, Gastrointestinal Symptom Rating Scale; RPR, rapid plasma reagin.

### Statistics

Quantitative parameters were presented as a median (interquartile range). Comparison of categorical data was carried out by the Fisher exact test, and the comparison of quantitative and semi-quantitative data was evaluated by the Mann–Whitney method. Changes in the values of extended and semi-quantitative parameters were assessed by the Wilcoxon test. Significance criterion was *P* < 0.05. Statistical data processing was carried out using the STATISTICA 10 software (StatSoft Inc, Tulsa, K). Effectiveness of the therapy was assessed according to the per-protocol analysis, and safety and compliance were assessed according to the intention-to-treat analysis.

### Ethics approval and consent to participate

This study was approved by the Local ethics committee of Sechenov University (protocol № 10–19 dated July 17, 2019) in accordance with the Helsinki declaration. Informed consent was obtained from all subjects involved in the study.

## RESULTS

**Figure 1.**
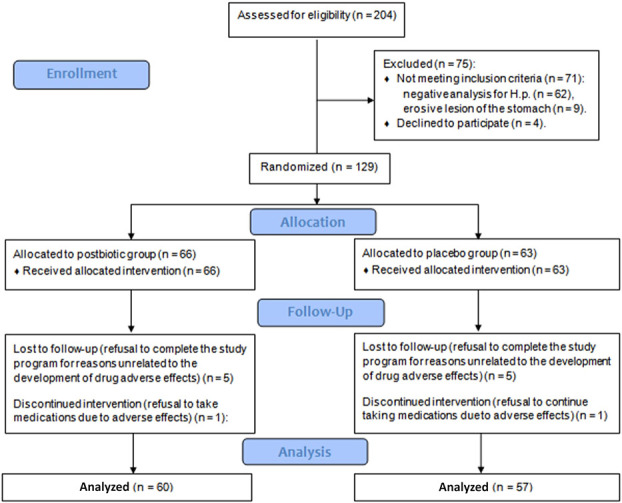
CONSORT 2010 flow diagram. H.p., *Helicobacter pylori*.

Of the 204 screened patients, 129 met the criteria and were included in the study. Among them, 66 were randomized to the postbiotic group and 63 to the placebo group. Five patients in each group refused to visit the centers again (visits 3 and/or 4) to evaluate the results of the therapy. One patient in each group developed adverse effects that led to the termination of eradication therapy before the minimum time (10 days), so they were also excluded from the analysis. In total, data from 60 patients in the postbiotic group and 57 patients in the placebo group were analyzed (Figure 1). There was no significant difference in baseline parameters between the 2 groups (Table 2). There was no significant difference between the groups in the incidence of comorbidities and the use of medication to treat them (Supplementary Table 1, Supplementary Digital Content 1, http://links.lww.com/CTG/B180).

**Table 2.** Patient baseline characteristics

	Postbiotic group (n = 60)	Placebo group (n = 57)	*P*
Age, yr	47 [38–56]	47 [36–53]	0.213
Male/female	19/41	21/36	0.346
Body mass index, kg/m^2^	24.8 [22.9–27.4]	25.6 [22.4–29.0]	0.343
Hemoglobin, 10^9^/L	137 [125–145]	138 [127–147]	0.278
White blood cell, 10^9^/L	6.1 [5.4–6.7]	6.1 [5.4–7.2]	0.332
Platelet, 10^9^/L	239 [213–283]	257 [216–296]	0.271
Erythrocyte sedimentation rate, mm/h	8 [6–12]	7 [4–10]	0.097
Serum total protein, g/L	72 [69–76]	72 [68–76]	0.983
Serum total bilirubin, µmol/L	10.3 [7.5–13.2]	10.6 [7.0–16.4]	0.691
Serum creatinine, µmol/L	82 [74–90]	79 [74–93]	0.775
Serum amylase, U/L	59 [42–113]	55 [44–78]	0.325
Serum alkaline phosphatase, U/L	110 [62–158]	106 [67–146]	0.907
Serum ALT, U/L	19 [13–25]	19 [15–28]	0.258
Serum AST, U/L	20 [16–26]	20 [17–24]	0.883
SF-36 questionnaire			
Physical functioning	90 [78–95]	85 [70–95]	0.347
Physical role functioning	75 [25–100]	75 [25–100]	0.633
Emotional role functioning	67 [0–100]	33 [33–100]	0.593
Vitality	55 [45–65]	55 [40–70]	0.972
Mental health	56 [44–68]	60 [48–72]	0.804
Social role functioning	75 [63–88]	75 [63–88]	0.699
Bodily pain	68 [45–78]	95 [45–78]	0.789
General health perceptions	55 [45–70]	50 [40–60]	0.198
7 × 7 questionnaire			
Pain in the stomach area	4 [3–5]	4 [2–5]	0.872
A burning sensation in the stomach area	3 [0–4]	2 [0–4]	0.531
Fullness in the stomach after a meal	2 [1–3]	2 [1–3]	0.624
Early satiety	2 [0–3]	2 [0–3]	0.610
Abdominal pain decreases after a bowel movement	0 [0–2]	0 [0–3]	0.762
Bloating	4 [0–5]	4 [0–5]	0.485
Abnormal stool consistency or frequency	0 [0–2]	0 [0–2]	0.825
Total	15 [11–19]	15 [11–20]	0.721
Gastrointestinal Symptom Rating Scale			
Upper abdominal pain or discomfort	4 [3–4]	4 [2–5]	0.543
Heartburn	2 [1–4]	2 [1–4]	0.777
Acid reflux	2 [1–4]	3 [1–4]	0.324
Hunger pains	2 [1–4]	2 [1–3]	0.627
Nausea	1 [1–3]	1 [1–3]	0.663
Rumbling	3 [2–4]	3 [2–5]	0.130
Bloating	4 [2–4]	4 [3–5]	0.103
Burping	3 [2–5]	3 [2–4]	0.379
Passing gas or flatus	4 [2–5]	3 [1–5]	0.612
Constipation	1 [1–3]	1 [1–3]	0.709
Diarrhea	1 [1–2]	1 [1–3]	0.612
Loose stools	1 [1–2]	1 [1–3]	0.281
Hard stools	1 [1–3]	1 [1–3]	0.693
Urgent need to have a bowel movement	1 [1–2]	1 [1–2]	0.563
Sensation of not completely emptying the bowels	2 [1–3]	1 [1–3]	0.403
Total	34 [26–47]	33 [27–53]	0.576

ALT, alanine transaminase; AST, aspartate aminotransferase.

At the end of the follow-up period, 2 patients (3.3%) in the postbiotic group and 8 patients (14.0%) in the placebo group had a positive 13C-UBT for *H. pylori*. The eradication efficiency was 96.7% for the postbiotic group and 86.0% for the placebo group (*P* = 0.039; Table 3).

**Table 3.** Efficiency of *Helicobacter pylori* eradication in patients receiving the postbiotic or placebo as an adjuvant to eradication therapy

	Eradication is effective	Eradication is not effective	Eradication rate	*P*
Postbiotic	58	2	97.3%	0.039
Placebo	49	8	86.0%	

By the end of the follow-up period, both groups showed significant improvements in the quality of life according to all sections of the SF-36 scale with no significant differences between groups (Table 4).

**Table 4.** Changes in digestive symptoms (decreases in the scores of the 7 × 7 and GSRSs) and quality of life (increases in the scores of the SF-36 scale) of the patients at the end of the eradication therapy (day 14) and at the end of the follow-up period (day 56 ± 3) compared with baseline

	At the end of eradication therapy			At the end of the follow-up period		
	Postbiotic group	Placebo group	*P*	Postbiotic group	Placebo group	*P*
SF-36 questionnaire						
Physical functioning	5 [0 to 10]^a^	0 [0 to 10]^a^	0.898	0 [0 to 10]^a,b^	5 [0 to 15]^a,b^	0.344
Physical role functioning	0 [0 to 25]	0 [0 to 25]	0.529	0 [0 to 50]^a,b^	0 [0 to 50]^a,b^	0.583
Emotional role functioning	0 [0 to 33]	0 [0 to 34]	0.293	0 [0 to 67]^a,b^	33 [0 to 67]^a,b^	0.414
Vitality	5 [0 to 10]^a^	5 [0 to 20]^a^	0.655	10 [0 to 20]^a,b^	10 [0 to 25]^a,b^	0.583
Mental health	4 [0 to 8]^a^	4 [0 to 12]^a^	0.915	8 [0 to 20]^a,b^	8 [4 to 16]^a,b^	0.894
Social role functioning	0 [0 to 25]^a^	0 [0 to 13]^a^	0.497	13 [0 to 25]^a,b^	13 [0 to 25]^a,b^	0.938
Bodily pain	10 [0 to 23]^a^	10 [0 to 22]^a^	0.943	22 [2 to 32]^a,b^	22 [0 to 42]^a,b^	0.940
General health perceptions	0 [−3 to 10]^a^	5 [−5 to 10]^a^	0.910	5 [−5 to 10]^a^	5 [0 to 15]^a^	0.400
7 × 7 questionnaire						
Pain in the stomach area	2 [1 to 3]^a^	2 [0 to 3]^a^	0.413	3 [2 to 4]^a,b^	3 [2 to 4]^a,b^	0.791
A burning sensation in the stomach area	1 [0 to 3]^a^	0 [0 to 2]^a^	0.879	2 [0 to 4]^a,b^	0 [0 to 3]^a,b^	0.436
Fullness in the stomach after a meal	1 [0 to 2]^a^	1 [0 to 2]^a^	0.711	1 [0 to 2]^a,b^	2 [0 to 3]^a,b^	0.629
Early satiety	0 [0 to 2]^a^	0 [0 to 2]^a^	0.541	1 [0 to 3]^a,b^	1 [0 to 3]^a,b^	0.934
Abdominal pain decreases after a bowel movement	0 [0 to 1]	0 [0-0]	0.378	0 [0 to 2]^a^	0 [0 to 2]^a,b^	0.954
Bloating	1 [0 to 2]^a^	2 [0 to 3]^a^	0.436	1 [0 to 3]^a,b^	2 [0 to 4]^a,b^	0.476
Abnormal stool consistency or frequency	0 [0-0]	0 [0-0]	0.943	0 [0 to 1]^b^	0 [0 to 1]^b^	0.380
Total	5 [2 to 9]^a^	7 [3 to 11]^a^	0.432	9 [6 to 13]^a,b^	10 [6 to 15]^a,b^	0.536
GSRS						
Upper abdominal pain or discomfort	1 [0 to 2]^a^	1 [0 to 2]^a^	0.250	2 [0 to 3]^a,b^	2 [1 to 3]^a,b^	0.817
Heartburn	0 [0 to 2]^a^	0 [0 to 2]^a^	0.357	1 [0 to 2]^a,b^	1 [0 to 2]^a^	0.795
Acid reflux	0 [0 to 2]^a^	0 [0 to 1]^a^	0.501	0 [0 to 2]^a,b^	1 [0 to 2]^a,b^	0.260
Hunger pains	0 [0 to 2]^a^	0 [0 to 2]^a^	0.155	1 [0 to 2]^a,b^	0 [0 to 2]^a^	0.866
Nausea	0 [0 to 1]^a^	0 [0 to 1]^a^	0.460	0 [0 to 2]^a,b^	0 [0 to 2]^a,b^	0.978
Rumbling	1 [0 to 2]^a^	1 [0 to 2]^a^	0.078	1 [0 to 2]^a,b^	2 [0 to 3]^a,b^	0.141
Bloating	1 [0 to 2]^a^	2 [0 to 2]^a^	0.108	1 [0 to 3]^a,b^	2 [1 to 4]^a,b^	0.059
Burping	1 [0 to 2]^a^	1 [0 to 2]^a^	0.301	1 [0 to 2]^a,b^	2 [0 to 3]^a,b^	0.068
Passing gas or flatus	0 [0 to 2]^a^	1 [0 to 2]^a^	0.567	1 [0 to 2]^a,b^	1 [0 to 3]^a,b^	0.834
Constipation	0 [0 to 1]^a^	0 [0-0]	0.343	0 [0 to 1]^a^	0 [0 to 1]^a^	0.851
Diarrhea	0 [−1 to 1]	0 [0 to 1]	0.351	0 [0 to 1]^a,b^	0 [0 to 1]^a,b^	0.657
Loose stools	0 [0 to 1]	0 [0 to 1]	0.249	0 [0 to 1]^a,b^	0 [0 to 2]^a,b^	0.105
Hard stools	0 [0 to 1]^a^	0 [0 to 1]^a^	0.806	0 [0 to 1]^a^	0 [0 to 1]^a^	0.965
Urgent need to have a bowel movement	0 [0-0]	0 [0 to 1]	0.406	0 [0-0]	0 [0 to 1]	0.151
Sensation of not completely emptying the bowels	0 [0 to 1]^a^	0 [0 to 1]^a^	0.494	0 [0 to 2]^a^	0 [0 to 1]^a^	0.271
Total	7 [1 to 15]^a^	7 [1 to 18]^a^	0.257	11 [4 to 21]^a,b^	16 [5 to 25]^a,b^	0.349

GSRS, Gastrointestinal Symptom Rating Scale.

a Significant changes compared with the baseline (the Wilcoxon test).

b Significant changes at the end of the follow-up period compared with the end of eradication therapy (the Wilcoxon test).

At the end of the eradication therapy (Day 14) in both groups, there were significant improvements compared with baseline in most digestive symptoms, including symptoms of FD (pain in the stomach area, a burning sensation in the stomach area, fullness in the stomach after a meal, early satiety, and bloating), which became even more significant by the end of the follow-up period (Table 4). There was no significant difference in the change in the severity of digestive symptoms neither at the end of the eradication therapy nor at the end of the follow-up period between the groups (Table 4).

There was no significant change in any of the tested laboratory parameters from baseline to the end of eradication therapy, and there were also no differences in these parameters between the groups (Supplementary Table 2, Supplementary Digital Content 1, http://links.lww.com/CTG/B180).

Compliance with the postbiotic was 100% in 98.5% of patients and did not significantly differ from the compliance with placebo. Compliance with standard eradication therapy was 100% in 97.0% of patients in the postbiotic group and did not differ significantly from that in the placebo group (Table 5).

**Table 5.** Compliance with intervention

Compliance	Postbiotic group (n = 66)	Placebo group (n = 63)	*P*
Compliance with postbiotic/placebo			
100%, n (%)	65 (98.5%)	62 (98.4%)	0.736
76–99%, n (%)	—	1 (1.6%)	
50–75%, n (%)	1 (1.5%)	—	
<50%, n (%)	—	—	
Compliance with esomeprazole			
100%, n (%)	65 (98.5%)	62 (98.4%)	0.736
76–99%, n (%)	—	1 (1.6%)	
50–75%, n (%)	1 (1.5%)	—	
<50%, n (%)	—	—	
Compliance with amoxicillin			
100%, n (%)	64 (97.0%)	60 (95.2%)	0.478
76–99%, n (%)	1 (1.5%)	2 (3.2%)	
50–75%, n (%)	—	1 (1.6%)	
<50%, n (%)	1 (1.5%)	—	
Compliance with clarithromycin			
100%, n (%)	64 (97.0%)	60 (95.2%)	0.478
76–99%, n (%)	1 (1.5%)	2 (3.2%)	
50–75%, n (%)	—	1 (1.6%)	
<50%, n (%)	1 (1.5%)	—	

We found that 30.3% of patients in the postbiotic group and 38.0% of patients in the placebo group developed adverse effects. Most of them were mild. Only in 1.5% of patients in the postbiotic group and in 1.6% of patients in the placebo group, the development of adverse effects required discontinuation of drugs (pain in the lumbar region in one patient in the postbiotic group; severe nausea with bitter taste in the mouth in 1 patient in the placebo group). Serious adverse effects were not registered. The postbiotic regimen tended to cause diarrhea less frequently than the placebo, but this difference did not reach the level of significance (10.6% vs 15.9%; *P* = 0.267). However, the overall number of digestive adverse effects in the postbiotic group was significantly lower than in the placebo group. The incidence of nondigestive adverse effects and laboratory abnormalities did not significantly differ between the groups (Table 6).

**Table 6.** Adverse effects of the therapy

	Postbiotic group (n = 66)	Placebo group (n = 63)	*P*
Patients with any adverse effects, n (%)	20 (30.3%)	24 (38.0%)	0.228
Patients with mild adverse effects, n (%)	14 (21.2%)	19 (30.2%)	0.168
Patients with moderate adverse effects, n (%)	6 (9.1%)	5 (7.9%)	0.533
Patients with severe adverse effects, n (%)	—	—	
Patients with adverse effects not requiring intervention, n (%)	18 (27.3%)	21 (33.3%)	0.289
Patients with adverse effects leading to drug withdrawal, n (%)	1 (1.5%)	1 (1.6%)	0.740
Patients with adverse effects leading to the prescription of corrective drugs, n (%)	2 (3.0%)	3 (4.8%)	0.478
Distinct adverse effects			
Digestive			
Bitterness in the mouth, n (%)	12 (18.2%)	14 (22.2%)	0.362
Diarrhea, n (%)	7 (10.6%)	10 (15.9%)	0.267
Constipation, n (%)	—	1 (1.6%)	0.488
Nausea, n (%)	3 (4.5%)	5 (7.9%)	0.333
Abdominal pain, n (%)	1 (1.5%)	3 (4.8%)	0.478
Gurgling in the stomach, n (%)	—	1 (1.6%)	0.488
Flatulence, n (%)	1 (1.5%)	—	0.512
Belching, n (%)	1 (1.5%)	—	0.512
Total cases of digestive symptoms, n	**25**	**34**	**0.049**
Nondigestive			
Headache, n (%)	—	1 (1.6%)	0.488
Dizziness, n (%)	—	1 (1.6%)	0.488
Pain in the lumbar region, n (%)	1 (1.5%)	—	0.512
Dry mouth, n (%)	1 (1.5%)	2 (3.2%)	0.482
Burning tongue, n (%)	1 (1.5%)	—	0.512
Multiple aphthae on the tongue and gums, n (%)	1 (1.5%)	—	0.512
Total cases of nondigestive symptoms, n	**4**	**4**	**0.615**
Laboratory			
Increased serum creatinine level, n (%)	1 (1.5%)	—	0.512
Increased serum ALT level, n (%)	2 (3.0%)	2 (3.2%)	0.673
Total cases of laboratory abnormalities, n	**3**	**2**	**0.803**

ALT, alanine transaminase. Subtotal highlighted in bold.

## DISCUSSION

*H. pylori* infection is one of the most common human infections in the world. This bacterium is recognized as the etiological agent of most forms of chronic gastritis and peptic ulcer, and is also involved in the pathogenesis of gastric cancer and mucosa-associated lymphoid tissue lymphoma (6). It has also been suggested that it is responsible for the development of some cases of FD. The eradication therapy in such patients can lead to a regression of symptoms (6), which coincides with the results of our study.

Therefore, the eradication of *H. pylori* becomes an important target. Several drug regimens have been proposed, among which those with proton-pump inhibitor + amoxicillin + clarithromycin is one of the first-line regimens in countries with low resistance of *H. pylori* to clarithromycin (6). Recent studies have shown the benefit of longer therapy (14 days vs 10 and 7 days (6)). However, even in this case, the effectiveness of eradication therapy was about 80%–90% (6,15,16,21,22). To further increase it, several methods have been proposed, including the use of probiotics (23). Among several microorganisms tested for this purpose, one of the most interesting is *L. reuteri* that survives in the gastric acid environment, colonizes the gastric mucosa, and inhibits the growth of several pathogenic bacteria, including *H. pylori* (24). Some strains of this bacterium have shown the ability to reduce the load of *H. pylori* (decrease in 13C-UBT values) (25,26). The use of this probiotic together with proton-pump inhibitor led to the eradication of *H. pylori* in a number of patients (25,26). However, despite the fact that the efficacy of 10–14-day first-line eradication therapy combined with this probiotic in many studies tended to be higher than without it (27–31), this difference reached significance only in the most recent study from Malaysia (32).

The efficacy of nonviable *L. reuteri* (postbiotic) for this purpose was also analyzed. After long-term screening, the *DSM*17648, a strain with maximum anti-Helicobacter activity in artificial gastric juice, was selected. Its mechanism of action is to bind to *H. pylori* and reducing mobility and adhesion ability of these pathogenic bacteria (10). Postbiotic based on this strain has been marketed under the trade name Pylopass. It showed the ability to reduce *H. pylori* load in the stomach according to the UBT (10,^13^,14), and its efficacy for *H. pylori* eradication in combination with IPP was as effective as standard eradication therapy (33). The Maastricht VI/Florence consensus mentions nonviable *L. reuteri DSM*17648 as one of the beneficial microorganisms shown to be effective in reducing gastrointestinal side effects caused by *H. pylori* eradication therapies, reducing abdominal complaints and seeming to have the potential to induce a significant faster recovery of gastrointestinal microbiota (6).

Our study is the first multicenter placebo-controlled study to confirm the efficacy of nonviable *L. reuteri DSM*17648 (Pylopass) as an enhancer of standard 14-day first-line bismuth-free eradication therapy in adults.

A recent clinical study by Yang et al (34) conducted in China did not show the effect of Pylopass on the eradication efficiency. One explanation for it could be the difference in therapy regimens and dosage forms. Yang et al used a 14-day pretreatment with nonviable *L. reuteri DSM*17648 with a 14-day cotreatment (eradication therapy + nonviable *L. reuteri DSM*17648), whereas we used the same cotreatment with a 14-day post-treatment. In addition, the patients in China administered Pylopass in a form of loose powder, and not in encapsulated form, as the patients in our study. We should also note the seemingly higher compliance to the medication in our study, with 100% compliance in more than 95% of patients in both groups, whereas in the study of Yang et al (34) compliance over 80% was registered in 88,9% of patients in the postbiotic group and 80,6% patients in the placebo group. All this could lead to an insufficient effect of Pylopass on the *H. pylori* eradication in the study of Yang et al . Other reasons may be genetic differences between the patient cohorts and *H. pylori* strain differences, including antibiotic resistance, in geographically distinct populations. Further RCTs comparing treatment regimens and geographical locations are required to verify this hypothesis.

Although we did not obtain a significant reduction in the frequency of distinct adverse effects as a result of the addition of postbiotic *L. reuteri DSM*17648 to the eradication therapy, the overall number of digestive adverse effects in the postbiotic group was significantly lower (*P* = 0.049). Yang et al (34) observed a significant effect from nonviable *L. reuteri DSM*17648 supplementation on the reduction of the incidence of eradication therapy-induced diarrhea (*P* = 0.022) and abdominal distention (*P* = 0.022). This may be due to the larger number of enrolled patients (n = 200 in (34) vs n = 129 in our study) and/or differences in the patient cohorts in these 2 studies. The study of Yang et al (34) was single-centered, with ethnically homogenous patient cohort, while our study was conducted in multiple independent locations. In addition, the study of Yang et al (34) included patients with *H. pylori* infection with no regard to their gastrointestinal symptoms. Our study was conducted under more stringent inclusion criteria, including only *H. pylori-*positive patients suffering from FD. We suppose that these initial differences in patients' symptomatic and/or their genetic features together with geographic diversity in microbiota composition could also explain for the variabilities in the observed results.

The strength of this study is its design (multicenter, randomized, double-blinded, placebo-controlled), which is the gold standard of clinical research. It is the first study showing that addition of inactivated *L. reuteri DSM*17648 to 14-day first-line eradication therapy significantly increases eradication efficacy.

One limitation of this study is the lack of a complete placebo group, consisting of patients receiving placebo instead of eradication therapy. This does not allow a correct assessment of the effect of the eradication therapy itself on the symptoms of FD. It would also be promising to evaluate how nonviable *L. reuteri DSM*17648 affect the efficacy of bismuth eradication quadruple therapy, which is another aim for future research.

The postbiotic based on *L. reuteri DSM*17648 significantly improves the efficacy of *H. pylori* eradication therapy in FD and decreases overall number of digestive adverse effects of this therapy.

## CONFLICTS OF INTEREST

**Guarantor of the article:** Roman Maslennikov, PhD.

**Specific author contributions:** V.I.: Research idea. V.I., I.M., E.P., and A.S.: Study design. All authors: Research and data analysis. R.M.: Draft writing. All authors: Draft editing.

**Financial support:** The study was sponsored by Novozymes A/S (Denmark) and Parusin (Germany). The sponsors had no role in the design of the study; in the collection, analyses, or interpretation of data; in the writing of the manuscript; or in the decision to publish the results.

**Potential competing interests:** The study was sponsored by Novozymes A/S (Denmark) and Parusin (Germany). The sponsors had no role in the design of the study; in the collection, analyses, or interpretation of data; in the writing of the manuscript; or in the decision to publish the results.

**Study Highlights**

**WHAT IS KNOWN**

✓ The effectiveness of eradication of *Helicobacter pylori* infection is insufficient.

**WHAT IS NEW HERE**

✓ The postbiotic improves the effectiveness of *H. pylori* eradication.
✓ The postbiotic decreases the overall number of digestive adverse effects of eradication therapy.

## Supplementary Material

## References

[1] Stanghellini V Chan FCL Hasler WL Gastroduodenal disorders. Gastroenterology 2016;150(6):1380–92. 27147122 10.1053/j.gastro.2016.02.011

[2] Ivashkin VT Mayev IV Sheptulin AA Diagnosis and treatment of the functional dyspepsia: Clinical guidelines of the Russian Gastroenterological Association. Russ J Gastroenterol Hepatol Coloproctol 2017;27(1):50–61.

[3] Talley N. Functional dyspepsia: Advances in diagnosis and therapy. Gut liver 2017;11(3):349–57. 28452210 10.5009/gnl16055 PMC5417776

[4] Madisch A Andresen V Enck P The diagnosis and treatment of functional dyspepsia. Dtsch Arztebl Int 2018;115(13):222–32. 29669681 10.3238/arztebl.2018.0222 PMC5938438

[5] Diaconu S Predescu A Moldoveanu A *Helicobacter pylori* infection: Old and new. J Med Life 2017;10(2):112–7. 28616085 PMC5467250

[6] Malfertheiner P Megraud F Rokkas T Management of *Helicobacter pylori* infection: The Maastricht VI/Florence consensus report. Gut 2022;71(9):1724–62. 10.1136/gutjnl-2022-327745 35944925

[7] Ivashkin VT Lapina TL Maev IV, et al. Clinical practice guidelines of Russian Gastroenterological Association, Scientific Society for the Clinical Study of Human Microbiome, Russian Society for the Prevention of Non-Communicable Diseases, Interregional Association for Clinical Microbiology and Antimicrobial Chemotherapy for *H. pylori* diagnostics and treatment in adults. Russ J Gastroenterol Hepatol Coloproctol. 2022;32(6):72–93.

[8] Ivashkin VT Maev IV Abdulganieva DI, et al. Practical recommendations of Scientific Society for the Study of Human Microbiome and the Russian Gastroenterological Association on use of probiotics, prebiotics, synbiotics and functional foods in treatment and prevention of gastroenterological diseases in children and adults. Russ J Gastroenterol Hepatol Coloproctol. 2021;31(2):65–91.

[9] Lv Z Wang B Zhou X Efficacy and safety of probiotics as adjuvant agents for *Helicobacter pylori* infection: A meta-analysis. Exp Ther Med 2015;9(3):707–16. 25667617 10.3892/etm.2015.2174 PMC4316960

[10] Holz C Busjahn A Mehling H Significant reduction in *Helicobacter pylori* load in humans with non-viable *Lactobacillus reuteri DSM*17648: A pilot study. Probiotics Antimicrob Proteins 2015;7(2):91–100. 25481036 10.1007/s12602-014-9181-3 PMC4415890

[11] Shenderov BA Sinitsa AV Zakharchenko MM, et al. Metabiotics: Present State, Challenges and Perspectives. Switzerland: Springer International Publishing; 2020.

[12] Salminen S Collado MC Endo A The International Scientific Association of probiotics and prebiotics (ISAPP) consensus statement on the definition and scope of postbiotics. Nat Rev Gastroenterol Hepatol 2021;18(9):649–67. 33948025 10.1038/s41575-021-00440-6 PMC8387231

[13] Mehling H Busjahn A. Non-viable *Lactobacillus reuteri DSMZ* 17648 (Pylopass) as a new approach to *Helicobacter pylori* control in humans. Nutrients 2013;5(8):3062–73. 23917169 10.3390/nu5083062 PMC3775242

[14] Buckley M Lacey S Doolan A The effect of *Lactobacillus reuteri* supplementation in *Helicobacter pylori* infection: A placebo-controlled, single-blind study. BMC Nutr 2018;4:48. 32153909 10.1186/s40795-018-0257-4 PMC7050722

[15] Zagari RM Bianchi-Porro G Fiocca R Comparison of 1 and 2 weeks of omeprazole, amoxicillin and clarithromycin treatment for *Helicobacter pylori* eradication: The HYPER Study. Gut 2007;56(4):475–9. 17028126 10.1136/gut.2006.102269 PMC1856863

[16] De Francesco V Ridola L Hassan C Two-week triple therapy with either standard or high-dose esomeprazole for first-line *H. pylori* eradication. J Gastrointestin Liver Dis 2016;25(2):147–50. 27308644 10.15403/jgld.2014.1121.252.2w3

[17] Bordin DS Voynovan IN Khomeriki SG Efficacy and safety of *L. reuteri DSMZ*17648 in *Helicobacter pylori*-infected patients without absolute indications for eradication therapy. Lech vrach 2016;5:1–6.

[18] Ivashkin V Sheptulin A Shifrin O Clinical validation of the “7 × 7” questionnaire for patients with functional gastrointestinal disorders. J Gastroenterol Hepatol 2019;34(6):1042–8. 30462850 10.1111/jgh.14546

[19] Revicki DA Wood M Wiklund I Reliability and validity of the gastrointestinal symptom rating scale in patients with gastroesophageal reflux disease. Qual Life Res 1997;7(1):75–83. 10.1023/a:1008841022998 9481153

[20] Weinberger M Samsa GP Hanlon JT An evaluation of a brief health status measure in elderly veterans. J Am Geriatr Soc 1991;39(7):691–4. 2061535 10.1111/j.1532-5415.1991.tb03623.x

[21] Nyssen OP Bordin D Tepes B European Registry on *Helicobacter pylori* management (Hp-EuReg): Patterns and trends in first-line empirical eradication prescription and outcomes of 5 years and 21 533 patients. Gut 2021;70(1):40–54. 32958544 10.1136/gutjnl-2020-321372

[22] Bordin DS Embutnieks YV Vologzhanina LG European Registry on the management of *Helicobacter pylori* infection (Hp-EuReg): Analysis of 2360 patients receiving first-line therapy in Russia. Ter Arkh 2018;90(2):35–42. 10.26442/terarkh201890235-42 30701770

[23] Zhang M Zhang C Zhao J Meta-analysis of the efficacy of probiotic-supplemented therapy on the eradication of *H. pylori* and incidence of therapy-associated side effects. Microb Pathog 2020;147:104403. 32707316 10.1016/j.micpath.2020.104403

[24] Dargenio C Dargenio VN Bizzoco F *Limosilactobacillus reuteri* strains as adjuvants in the management of *Helicobacter pylori* infection. Medicina (Kaunas) 2021;57(7):733. 34357014 10.3390/medicina57070733 PMC8306855

[25] Dore MP Cuccu M Pes GM *Lactobacillus reuteri* in the treatment of *Helicobacter pylori* infection. Intern Emerg Med 2014;9(6):649–54. 24178436 10.1007/s11739-013-1013-z

[26] Francavilla R Lionetti E Castellaneta SP Inhibition of *Helicobacter pylori* infection in humans by *Lactobacillus reuteri* ATCC 55730 and effect on eradication therapy: A pilot study. Helicobacter 2008;13(2):127–34. 18321302 10.1111/j.1523-5378.2008.00593.x

[27] Dore MP Bibbò S Pes GM Role of probiotics in *Helicobacter pylori* eradication: Lessons from a study of *Lactobacillus reuteri* strains *DSM* 17938 and ATCC PTA 6475 (Gastrus®) and a proton-pump inhibitor. Can J Infect Dis Med Microbiol 2019;2019:3409820. 31065301 10.1155/2019/3409820 PMC6466906

[28] Emara MH Mohamed SY Abdel-Aziz HR. *Lactobacillus reuteri* in management of *Helicobacter pylori* infection in dyspeptic patients: A double-blind placebo-controlled randomized clinical trial. Therap Adv Gastroenterol 2014;7(1):4–13. 10.1177/1756283X13503514 PMC3871281 24381643

[29] Naghibzadeh N Salmani F Nomiri S Investigating the effect of quadruple therapy with *Saccharomyces boulardii* or *Lactobacillus reuteri* strain (*DSMZ* 17648) supplements on eradication of *Helicobacter pylori* and treatments adverse effects: A double-blind placebo-controlled randomized clinical trial. BMC Gastroenterol 2022;22(1):107. 35255819 10.1186/s12876-022-02187-z PMC8903632

[30] Francavilla R Polimeno L Demichina A . *Lactobacillus reuteri* strain combination in *Helicobacter pylori* infection: A randomized, double-blind, placebo-controlled study. J Clin Gastroenterol 2014;48(5):407–13. 24296423 10.1097/MCG.0000000000000007

[31] Poonyam P Chotivitayatarakorn P Vilaichone RK. High effective of 14-day high-dose PPI- bismuth-containing quadruple therapy with probiotics supplement for *Helicobacter pylori* eradication: A double blinded-randomized placebo-controlled study. Asian Pac J Cancer Prev 2019;20(9):2859–64. 31554388 10.31557/APJCP.2019.20.9.2859 PMC6976817

[32] Ismail NI Nawawi KNM Hsin DCC Probiotic containing *Lactobacillus reuteri DSM* 17648 as an adjunct treatment for *Helicobacter pylori* infection: A randomized, double-blind, placebo-controlled trial. Helicobacter 2023;28(6):e13017. 37614081 10.1111/hel.13017

[33] Muresan IAP Pop LL Dumitrascu DL. *Lactobacillus reuteri* versus triple therapy for the eradication of *Helicobacter pylori* in functional dyspepsia. Med Pharm Rep 2019;92(4):352–5. 31750434 10.15386/mpr-1375 PMC6853040

[34] Yang C Liang L Lv P Effects of non-viable *Lactobacillus reuteri* combining with 14-day standard triple therapy on *Helicobacter pylori* eradication: A randomized double-blind placebo-controlled trial. Helicobacter 2021;26(6):e12856. 34628695 10.1111/hel.12856

