# Supplementary material for: Efficacy and Safety of Postbiotic Contained Inactivated Lactobacillus reuteri (Limosilactobacillus reuteri) DSM17648 as Adjuvant Therapy in the Eradication of Helicobacter pylori in Adults With Functional Dyspepsia: A Randomized Double-Blind Placebo-Controlled Trial
Source: Clin Transl Gastroenterol. 2024 Jul 18;15(9):e1. doi: 10.14309/ctg.0000000000000750 (PMC11421730; doi:10.14309/ctg.0000000000000750)
Supplement: Supplementary file 1 [file ct9-15-e1g-s001.doc]

Supplementary Table 1. Concomitant diseases and their therapy in patient groups

|  | Postbiotic group (n=60) | Placebo group (n=57) | p |
| --- | --- | --- | --- |
| Concomitant diseases | | | |
| Arterial hypertension, n (%) | 9 (15.0%) | 9 (15.8%) | 0.554 |
| Metabolically associated fatty liver disease, n (%) | 2 (3.3%) | 1 (1.8%) | 0.519 |
| Asthma, n (%) | 2 (3.3%) | 1 (1.8%) | 0.519 |
| Urolithiasis disease, n (%) | 2 (3.3%) | 0 | 0.261 |
| Gastroesophageal reflux, n (%) | 11 (18.3%) | 7 (12.3%) | 0.258 |
| Cholelithiasis, n (%) | 3 (5.0%) | 1 (1.8%) | 0.329 |
| Varicose veins of the lower extremities, n (%) | 0 | 1 (1.8%) | 0.487 |
| Treated cancer in the stable remission, n (%) | 1 (1.7%) | 2 (3.5%) | 0.481 |
| Irritable bowel syndrome, n (%) | 8 (13.3%) | 6 (10.5%) | 0.429 |
| Appendectomy, n (%) | 2 (3.3%) | 1 (1.8%) | 0.519 |
| Depression/neurotic disorder, n (%) | 5 (8.3%) | 1 (1.8%) | 0.116 |
| Functional diseases of the biliary system, n (%) | 3 (5.0%) | 2 (3.5%) | 0.525 |
| Diabetes, n (%) | 1 (1.7%) | 0 | 0.513 |
| Colon diverticula, n (%) | 2 (3.3%) | 0 | 0.261 |
| Chronic pancreatitis, n (%) | 0 | 1 (1.8%) | 0.487 |
| Benign paroxysmal positional vertigo, n (%) | 1 (1.7%) | 0 | 0.513 |
| Hypothyroidism, n (%) | 0 | 2 (3.5%) | 0.235 |
| Drugs used | | | |
| Angiotensin converting protein inhibitors / Angiotensin receptor blockers, n (%) | 6 (10.0%) | 6 (10.5%) | 0.582 |
| Ursodeoxycholic acid, n (%) | 4 (6.7%) | 2 (3.5%) | 0.364 |
| Inhaled glucocorticoids, n (%) | 2 (3.3%) | 0 | 0.261 |
| Calcium channel blockers, n (%) | 2 (3.3%) | 2 (3.5%) | 0.671 |
| Prokinetics, n (%) | 13 (21.7%) | 7 (12.3%) | 0.135 |
| Diosmectite, n (%) | 0 | 1 (1.8%) | 0.487 |
| Postmenopausal hormone replacement therapy, n (%) | 0 | 3 (5.3%) | 0.113 |
| Psyllum, n (%) | 1 (1.7%) | 0 | 0.513 |
| Diosmin, n (%) | 0 | 1(1.8%) | 0.487 |
| Lactulose, n (%) | 0 | 1(1.8%) | 0.487 |
| Tamoxifen, n (%) | 0 | 1(1.8%) | 0.487 |
| Beta blockers, n (%) | 2 (3.3%) | 2 (3.5%) | 0.671 |
| Diuretic, n (%) | 1 (1.7%) | 2 (3.5%) | 0.481 |
| Antidepressants and other psychotropic drugs, n (%) | 10 (16.7%) | 5 (8.8%) | 0.159 |
| Antispasmodics, n (%) | 6 (10.0%) | 5(8.8%) | 0.536 |
| Alginates, n (%) | 9 (15.0%) | 4 (7.0%) | 0.140 |
| Betahistine, n (%) | 1 (1.7%) | 0 | 0.513 |
| Thyroxine, n (%) | 0 | 2 (3.5%) | 0.235 |
| Carbamazepine, n (%) | 1 (1.7%) | 0 | 0.513 |
| Metformin, n (%) | 1 (1.7%) | 0 | 0.513 |
| Cholecalciferol, n (%) | 0 | 1(1.8%) | 0.487 |

Supplementary Table 2. Changes in the values of the main laboratory parameters as a result of eradication therapy (the difference between Visit 3 and Visit 1).

|  | Postbiotic group | Placebo group | p |
| --- | --- | --- | --- |
| Hemoglobin, 10^9^/L | 1[-3-5] | 1[-4-4] | 0.525 |
| White blood cell, 10^9^/L | 0.0[-0.8-0.7] | 0.0[-0.6-0.6] | 0.645 |
| Platelet, 10^9^/L | 0[-25-13] | 0[-23-14] | 0.697 |
| Erythrocyte sedimentation rate, mm/h | 0[-3-3] | 1[-1-4] | 0.256 |
| Serum total protein, g/L | 0[-2-2] | 0[-2-3] | 0.418 |
| Serum total bilirubin, µmol/L | -0.2[-2.3-3.9] | 0[-2.0-1.7] | 0.610 |
| Serum creatinine, µmol/L | 2[-8-8] | 0[-6-5] | 0.262 |
| Serum amylase, U/L | 2[-9-11] | 4[-4-14] | 0.295 |
| Serum alkaline phosphatase, U/L | 2[-16-30] | 0[-14-17] | 0.659 |
| Serum ALT, U/L | 4[-3-8] | 1[-2-6] | 0.294 |
| Serum AST, U/L | 2[-2-4] | 1[-2-4] | 0.902 |
